# Major improvement using peroperative 3D compared to 2D imaging for SI screw fixation in traumatic pelvic fractures

**DOI:** 10.1007/s00068-026-03215-3

**Published:** 2026-06-02

**Authors:** F. J. C. van Eerten, K. E. M. Benders, M. C. P. M. van Baal, F. Hietbrink

**Affiliations:** Department of Trauma Surgery, University Medical Center Utrecht, Utrecht, GA 3508 Netherlands

**Keywords:** Sacro-iliac screw, Pelvis, 3D imaging, Trauma

## Abstract

**Introduction:**

Pelvic fractures, though accounting for only 3% of skeletal injuries, carry a high mortality rate, primarily due to the associated risk of hemodynamic instability. Sacroiliac (SI) screw fixation is a common surgical intervention, which can be technically challenging due to the complex pelvic anatomy. Imaging techniques have evolved from 2D to 3D imaging for its improved accuracy in screw placement. The impact of this transition for SI screw placement was evaluated.

**Method:**

A single-center observational retrospective study was performed between 2013 and 2023. Patients *≥* 18 years who underwent SI screw placement following pelvic injuries were analyzed. 2D imaging was used until 2021, after which 3D imaging was implemented. Outcomes included the need for revision surgery during initial admission and < 18 months after trauma.

**Results:**

2D imaging was used in 42 patients and 3D imaging in 44 patients. Baseline characteristics and injury severity of both groups were comparable. In the 2D group, 18 patients (42.9%) had suboptimal placement of the SI screw. Of these, 3 (7.1%) required revision surgery due to malpositioned screws. In contrast, no patients (0.0%) in the 3D group had suboptimally positioned screws or underwent revision surgery, (*p* < 0.001, *p* = 0.22).

**Discussion:**

Intraoperative 3D imaging enhanced the accuracy of SI screw placement, leading to an encouraging reduction in suboptimally placed SI screws. This improvement contributed a trend of fewer secondary revision surgeries and complications. These results suggested benefits of applying 3D imaging broadly.

## Introduction

Pelvic fractures are relatively rare (3%) skeletal injuries, but are associated with a relatively high mortality rate due to exsanguiniation [1–4]. Road traffic accidents and falls are the leading causes of pelvic fractures [5].

In pelvic trauma, sacral fractures or disruptions of the sacroiliac (SI) joint can occur [6, 7]. These injuries typically result from axial loading forces and sudden rotational movements [7]. Tears in the posterior ligamentous structures surrounding the SI joint may lead to both rotational and vertical instability of the pelvic ring [8]. In cases of pelvic ring instability, surgical fixation is required [2].

SI joint fixation is achieved using SI screw fixation [6, 9]. SI screws are usually placed percutaneously after closed reduction. The main advantage of this technique is low rate of infection and bleeding [6, 9]. Correct SI joint fixation is a challenging procedure, due to variations in anatomical structures and frequently small osseus corridor diameters [10, 11].

The main structures that are at risk of iatrogenic injury include iliac and gluteal vessels, sympathetic nerve chain and the L5-S1 nerve roots [10, 12]. Moreover, proper alignment of the SI screw is essential to prevent secondary displacement, chronic pain and even disability resulting from iatrogenic nerve injury [9].

Historically surgeons relied solely on per-operative two-dimensional (2D) imaging. Recently, significant progress has been made in intra-operative imaging, including the development of three-dimensional (3D) imaging and even navigation [10]. Using a 60-second automated orbital scan, approximately 100 images are acquired and reconstructed into axial, sagittal, and coronal views, as well as 2D and 3D reformations [10]. The 3D reformations allow for more precise assessment of K-wire or SI screw positioning compared to conventional 2D imaging. Moreover, the 3D imaging provides the opportunity for intra-operative adjustments and thereby reducing the need for post-operative revisions and implant-related complications.

The goal is to prevent SI screw malpositioning that would require revision surgery. Therefore, this study aimed to evaluate screw misplacement rates and the need for secondary revision surgeries following SI screw placement using either 2D or 3D intra-operative imaging.

## Methods

### Study design

This retrospective cohort observational study was conducted at the University Medical Center Utrecht, between 2013 and 2023. Patients were identified using the Dutch National Trauma Registry (DNTR) [13]. All data were stored and processed in accordance with applicable privacy and ethical guidelines. Ethical approval was granted by the UMC Utrecht Medical Ethics Review Committee (reference: 25U-0085_QoPF).

### Patient selection

All patients aged *≥* 18 years who sustained pelvic fractures were identified in the DNTR using the Abbreviated Injury Scale (AIS) [14]. The AIS coding was performed by dedicated DNTR datamanagers. Patients with AIS codes indicating pelvic fractures were selected. Subsequently, patients who underwent SI screw placement were included in this study.

### Patient categorization

Patients were categorized based on whether 2D imaging or 3D intra-operative imaging was performed. The 3D imaging was introduced at this center on 19th of February 2021. Therefore, patients treated before February 19, 2021, were included in the 2D group, and those treated afterward were included in the 3D group. The operative report was used to verify whether 2D or 3D imaging was used.

### Patient and injury characteristics

Patient and injury characteristics were collected, including age, gender, ASA score, comorbidities, injury mechanism, Injury Severity Score (ISS), type of pelvic fracture classified by Young-Burgess classification, and concomitant injuries.

Comorbidities included osteoporosis, diabetes mellitus or a previous medical history of pelvic fractures. Injury mechanisms were were categorized as: high energy fall, low energy fall, traffic accident involving a motor vehicle, traffic accident involving a bicycles, traffic accident involving a pedestrians, and miscellaneous (entrapment under heavy objects).

Motorized traffic accidents were defined as accidents involving cars or motorcycles. Bicycle accidents included both standard bicycles and an e-bikes, reaching speeds up to 25 km/h.

### Outcomes

**Fig. 1:**
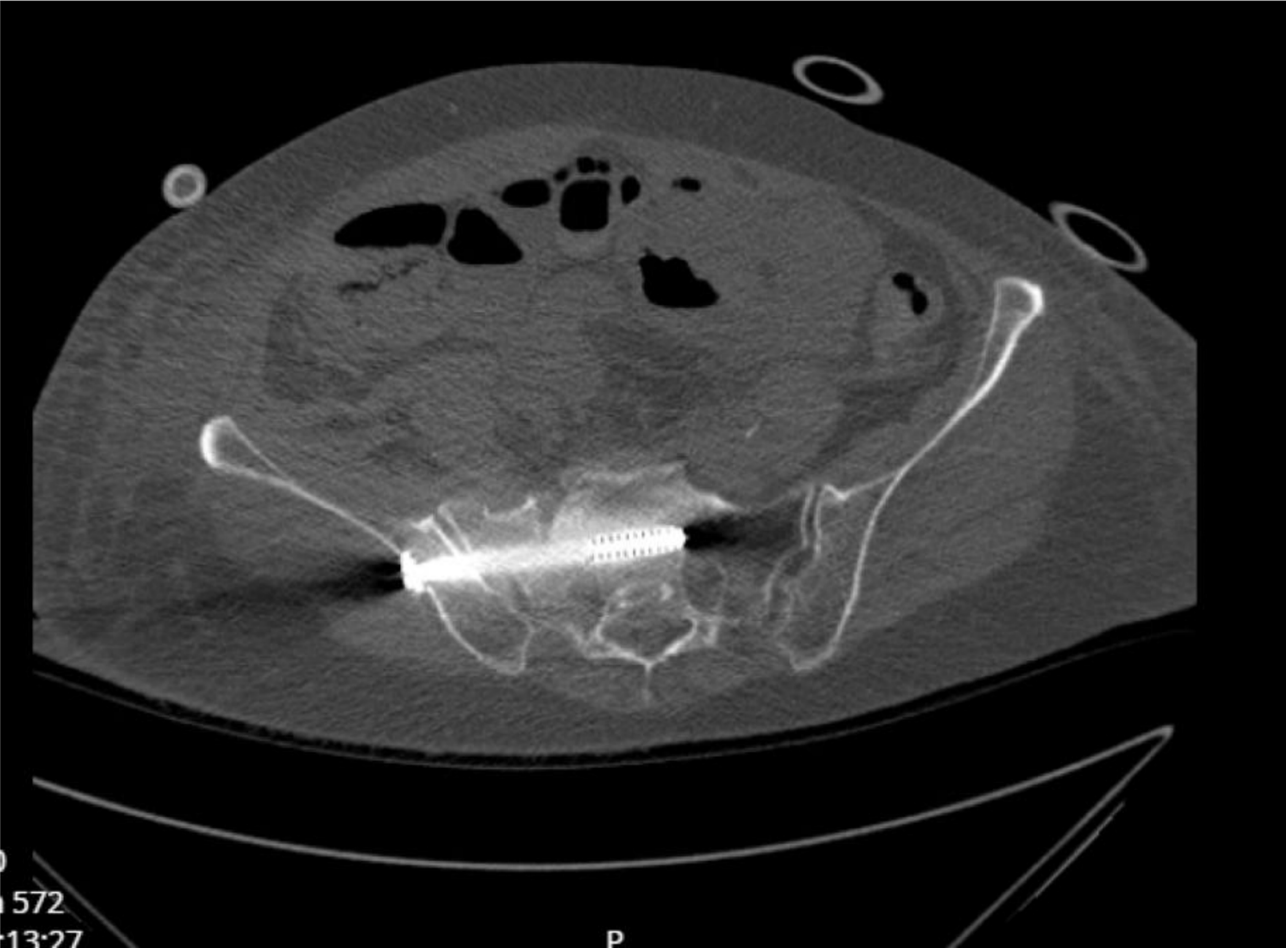
Example of a correctly placed SI screw. Axial view of CT scan. The SI screw is positioned in the cancellous bone, without traversing the neural foramen or anterior cortical protrusion

**Fig. 2:**
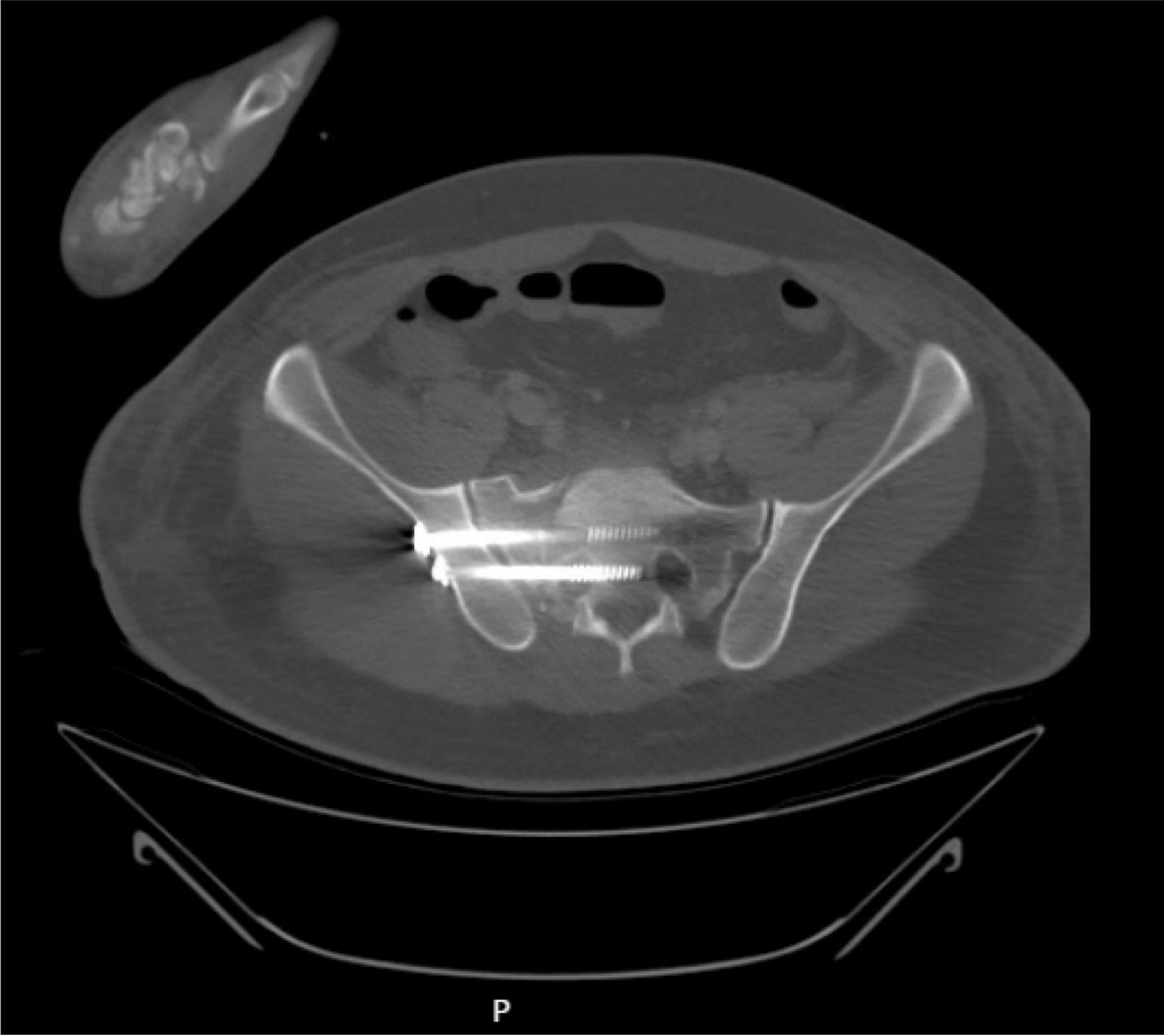
Example of a malpositioned SI screw. Axial view of CT scan. The posterior SI screw traverses the right neural foramen

Outcomes included suboptimal screw positioning and malposition. An example of a correctly placed SI screw is shown in Fig. 1, and an example of a malpositioned SI screw (traversing the neural foramen) is shown in Fig. 2. Suboptimal positioning was defined as a screw traversing the neural foramen, anterior cortical protrusion, or breach into the spinal canal or intervertebral L5/S1 disc space [15]. Malposition was defined as suboptimal positioning requiring revision surgery. Assessments were performed by two authors (FE and KB); in cases of disagreement, a third author (FH) was consulted.

It was also recorded whether adjustment of the K-wire or screw position was performed during the initial surgery or after postoperative CT scanning. Revision surgeries were categorized as occurring during initial admission, and within 1.5 years after traumatic injury. Complications included nerve injury and reasons for secondary procedures; malposition, nerve injury, infection, pseudoarthrosis, persistent pain complaints, secondary dislocation.

### Statistical analysis

Statistical analysis was performed using R for statistical computing (version 2024.12.0.46) [16]. Medians and Interquartile Ranges (IQR) were calculated for numerical variables and compared using Mann-Whitney-Wilcoxon test. Numbers and percentages were used to describe categorical variables and compared using the Chi-square test.

## Results

### Baseline characteristics

In 42 patients, SI screws were placed using 2D imaging, and in 44 patients, 3D imaging was used. Patient characteristics did not differ significantly in terms of age, gender, and ASA score (see Table 1).

**Table 1:**
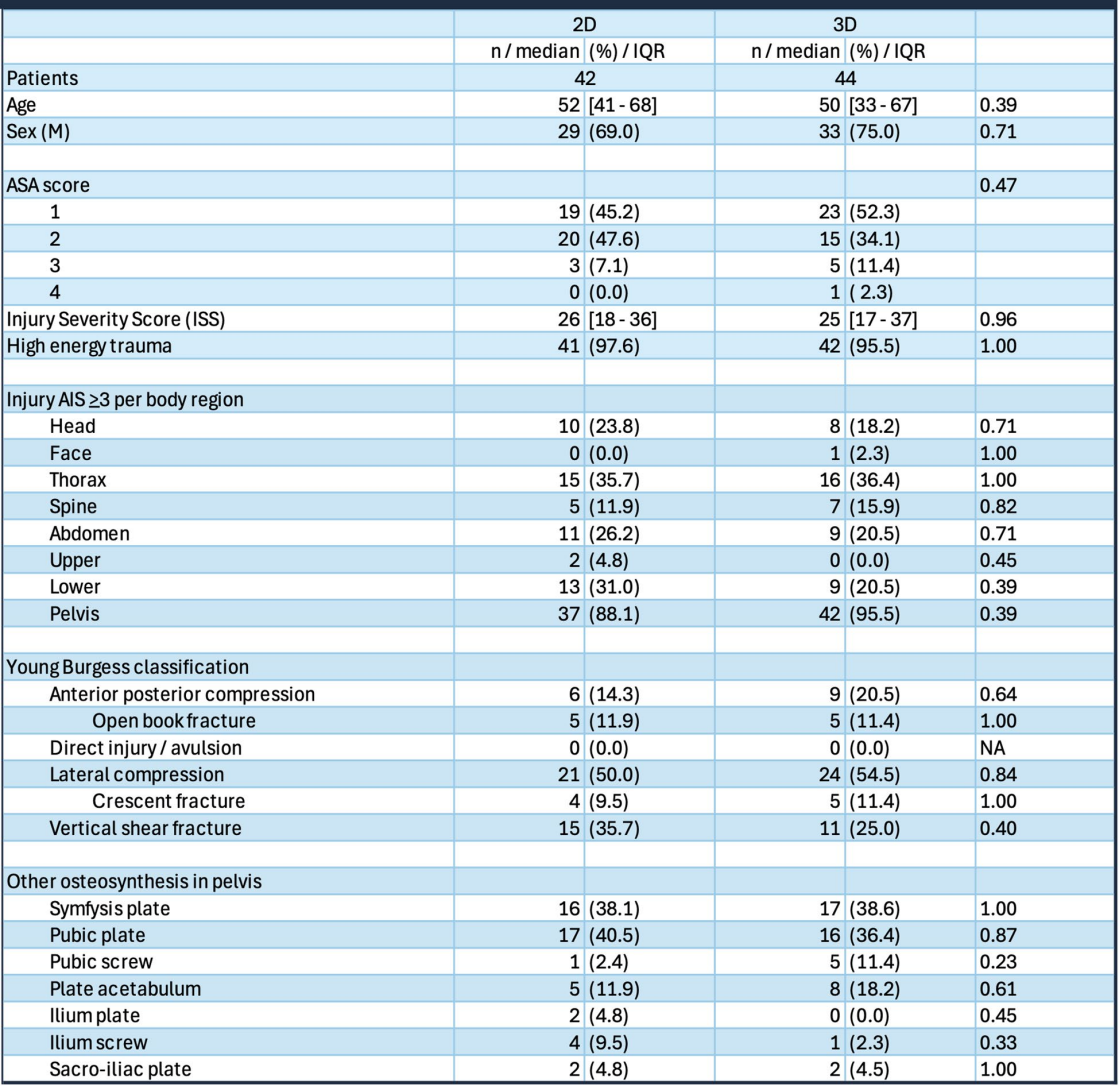
Baseline characteristics patients with a sacro-iliac (SI) screw

### Injury characteristics

The ISS in the 2D group was 26 [IQR 18–36], and in the 3D group it was 25 [IQR 17–37], *p* = 0.96. Most patients suffered from high-energy trauma; 41 (97.6%) in the 2D group and 42 (95.5%) in the 3D group, *p* = 1.00. Thoracic injuries were most concomitant in both groups; 15 (35.7%) in the 2D group and 16 (36.4%) in the 3D group, *p* = 1.00.

Regarding fracture patterns, approximately half of the patients sustained lateral compression injuries: 21 (50.0%) patients in the 2D group versus 24 (54.5%) patients in the 3D group, *p* = 0.84. The remaining patients predominantly suffered from vertical shear fractures; 15 (35.7%) patients in the 2D group, versus 11 (25.0%) in the 3D group, *p* = 0.40 (Table 1).

### Revision surgery

In the 3D group, 11 (25.0%) intra-operative adjustments of K-wire positioning were performed. Postoperative CT scanning was performed in all patients except one.

In total, 18 (42.9%) patients in the 2D group had a suboptimally placed SI screw on the postoperative CT scan, of whom 3 (7.1%) underwent revision surgery. In the 3D group, no patients had a malpositioned SI screw on the post-operative CT scan (0.0%; *p* < 0.001 and *p* = 0.22, respectively) (Table 2).

Within the first 1,5 years of follow-up, 5 (11.9%) patients in the 2D group and 3 (6.8%) patients in the 3D group (*p* = 0.72) underwent secondary procedures (Table 2). The most common reason for a secondary procedure was persistent pain in both the 2D and 3D group (Table 2).

Overall, revision surgery or a secondary procedure was required in 8 (19.0%) patients in the 2D group and in 3 (6.8%) in the 3D group (*p* = 0.24; Table 2).

**Table 2:**
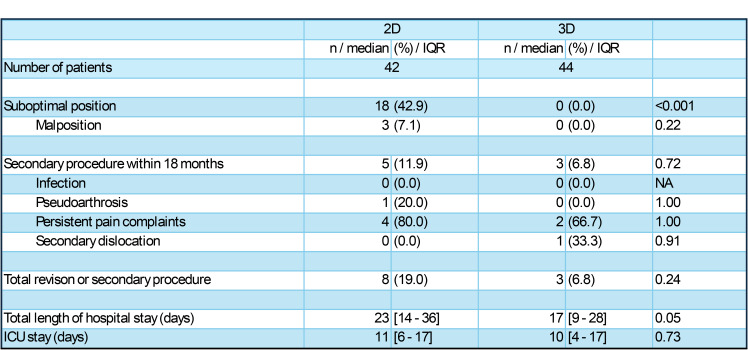
Outcomes of patients with a sacro-iliac (SI) screw

## Discussion

This study demonstrated that the use of 3D imaging substantially reduces the risk of suboptimally positioned SI screws, shown by the reduction from 43% to 0% subtoptimally placed SI screws and consequently, zero revisions within initial hospital admission in the 3D group.

### Difficulties in placing SI screw

Using 2D imaging remains technically challenging due to the complex anatomy of the pelvis [10]. Sacral dysmorphia, characterized by a narrow S1 canal, a steep alar slope, and a relatively high S2 body, has been reported in up to 15% of patients [11]. These variations complicate intraoperative imaging and screw trajectory planning, particularly when relying solely on 2D imaging, and especially in obese patients. In such cases, 3D imaging offers additional value by providing more precise spatial orientation and visualization of narrow safe corridors [10, 11]. Futhermore, 2D imaging is further complicated by osteophytes, spondylolistheses and excess intestinal gas [17].

The current study used 3D imaging in all patients, reducing potential bias. Intra-operative adjustments of K-wire placement were performed in 25% of patients in the 3D group, potentially preventing the need for secondary surgery. Furthermore, intraoperative 3D imaging promotes a more critical surgical approach by revealing opportunities for optimization. These findings suggest that the use of 3D imaging provides added value in all SI screw placements, not only in cases with dysmorphic pelvic anatomy.

### Radiation exposure in 2D and 3D imaging

Previous studies suggest that 3D imaging may reduce overall radiation exposure during SI screw placement. For example, Beck et al. reported a significantly lower radiation dose in the 3D group compared to the 2D group [18], due to higher images quality requiring fewer scans in the 3D versus 2D imaging group [18]. However, some ex vivo studies have found the opposite, with higher radiation associated with 3D imaging protocols [19–21]. This is because a single 3D scan involves a higher radiation load compared to one 2D scan [20]. However, fewer scans are typically required with 3D imaging than with 2D imaging, resulting in a reduced overall radiation load.

### Clinical relevance

Correct placement of SI screws is critical to avoid iatrogenic injury, particularly to neurovascular structures, and to ensure mechanical stability. Misplaced screws often necessitate revision surgery, which carries increased risks due to repeated anesthesia, prolonged operative time, and potentially higher radiation exposure [22, 23]. In this context, the use of 3D imaging may enhance surgical precision and reduce the risk of complications and revisions.

### Incidence of SI screw placement

It was observed that the number of SI screws increased over time, with 42 SI screw procedures performed in the period from 2013 to 2020 (8 years) and 44 in the period from 2021 to 2023 (3 years). The most likely explanations for this are that the referral of pelvic fractures became centralized during these years, and thus more pelvic fractures were treated in this level 1 trauma center. This includes all types of pelvic fractures. In addition, 3D imaging provides greater control over placement and improves surgical accuracy. This allows more team members to competently perform SI screw placement, rather than relying solely on dedicated surgeons.

### Limitations

This study has several limitations. First, the relatively small sample size limited the statistical power to confirm observed trends. However, the cohort size reflects the current clinical volume of patients treated with SI screw placement, and no additional comparable data were available for inclusion. Second, surgeon experience was not included as a variable in the analysis, despite its known influence on outcomes. Likely, in part due to increased centralization and the rising number of SI screw procedures, surgical expertise improved over time, which may have contributed to the observed better outcomes. At the same time, in the more recent period, SI screw procedures were performed by the entire trauma surgical team rather than a dedicated subgroup. Nevertheless, given the magnitude and abrupt nature of this improvement, the implementation of 3D imaging likely played a major role.

### The future of SI screw placement

Building on the transition from 2D to 3D imaging, real-time navigation can be incorporated. A previous systematic review by Thakkar et al. investigated fluoroscopy-based navigation [10]. To provide this intra-operative navigation, a fluoroscope is mounted on the C-arm, which tracks markers to determine their position relative to radiographic images [10]. This system provides real-time feedback and can be used in combination with 3D imaging [10]. Real-time verification of SI screw placement reduces operative time and decreases the need for post-placement imaging, thereby potentially lowering radiation exposure [10].

## Conclusion

This study demonstrated an encouraging reduction in suboptimally placed SI screws with the use of 3D imaging. This improvement may contribute to fewer complications and revision surgeries. These findings support the routine use of 3D imaging in all SI screw procedures.

## Data Availability

Restrictions apply to the availability of the data supporting the findings of this study, as they were used under license for the current study and are not publicly available. However, the data can be obtained from the authors upon reasonable request and with permission from University Medical Center Utrecht.
